# Triglyceride–glucose index and the risk of stroke in American adults: findings from the atherosclerosis risk in communities study

**DOI:** 10.1186/s13098-023-01161-3

**Published:** 2023-09-19

**Authors:** Xinyi Wang, Qiyu Liu, Tongxin Wang, Wende Tian, Xuanye Chen, Jie Zhang, Qiuyi Li, Dan Ma, Lin Zhao, Zhuo Chen, Hao Xu, Keji Chen

**Affiliations:** 1 National Clinical Research Center for Chinese Medicine Cardiology, Xiyuan Hospital, China Academy of Chinese Medical Sciences, No.1 XiYuan CaoChang, Haidian District, Beijing, 100091 China; 2 Graduate School, Beijing University of Chinese Medicine, No.11 Bei San Huan Dong Lu, Chaoyang District, Beijing, 100029 China; 3 Department of Integrative Cardiology, China-Japan Friendship Hospital, No.2 Yinghuayuan East Street, Chaoyang District, Beijing, 100029 China

**Keywords:** Insulin resistance, Triglyceride–glucose index, Stroke, Risk factors

## Abstract

**Objectives:**

The associations between the triglyceride–glucose (TyG) index with subsequent stroke in American adults are unknown. The aim of this study was to determine the associations between baseline and trajectories of TyG index with subsequent stroke in American adults.

**Methods:**

A total of 10,132 participants free of a history of stroke at baseline were included. We quantified the association of baseline and trajectories of TyG index with incident total stroke, ischemic stroke and intracerebral hemorrhage using Cox regression, restricted cubic splines and logistic regression analysis.

**Results:**

There were 909 incident stroke cases over a median follow-up of 26.6 years. After adjustment for potential confounders, each unit increase in the TyG index was associated with a 32.1% higher risk of incident stroke. Compared with participants in the lowest quartile of the baseline TyG index, those in the highest quartile had a greater risk of incident stroke [HR (95% CI) 1.254 (1.014–1.552)]. Restricted cubic splines showed that the risk of stroke increased in participants with a higher TyG index, especially when the TyG index was > 8.6. Results were similar for incident ischemic stroke. Compared with participants in the lowest quartile of the baseline TyG index, those in the second quartile had a lower risk of intracerebral hemorrhage [HR (95% CI) 0.494 (0.262–0.931)]. Five discrete trajectories with stable TyG indexes at various levels at follow-up visits were identified, and parallel results were observed for the associations of trajectories of TyG index with outcomes.

**Conclusions:**

The TyG index independently predict stroke progression.

**Supplementary Information:**

The online version contains supplementary material available at 10.1186/s13098-023-01161-3.

## Introduction

Stroke was the second-leading cause of death and the third-leading cause of death and disability in 2019 [1]. Each year, stroke affects about 795,000 individuals in the US, with many survivors experiencing persistent difficulty with daily tasks as a direct consequence [2]. The Global Burden of Disease Study found that 87.0% of the global burden of stroke was attributable to modifiable risk factors [1], suggesting the importance of early risk factor identification and control.

Insulin resistance (IR) is a hallmark of metabolic syndrome and is well-established as a critical risk factor for the occurrence and development of stroke [3]. The hyperinsulinemic–euglycemic clamp (HIEC) test is the gold standard test for insulin sensitivity. However, it is time-consuming, costly, and invasive, with limited applicability to research settings. The triglyceride–glucose (TyG) index includes fasting triglyceride and glucose and is a simple and reliable surrogate index for IR [4]. An elevated TyG index was associated with higher stroke risk in the Chinese population [5, 6]. The evidence suggests that the association between IR and stroke varies across ethnic groups [7]; nonetheless, few studies have investigated TyG index and stroke in the US, where an estimated 7.6 million (2.7%) Americans aged ≥ 20 years suffer strokes [8]. In addition, a previous study only used a single measure of TyG level and did not explore the relationship between longitudinal patterns of TyG and stroke events.

Therefore, the present study explored the influence of the baseline TyG index and different trajectories of its change over six visits on the risk of stroke and its subtypes using data from the Atherosclerosis Risk in Communities (ARIC) study in four US communities.

## Method

### Study population

The ARIC Study is a prospective cohort study initiated in 1987 to study cardiovascular disease risk factors and natural history. ARIC enrolled 15,792 participants from four US communities (Washington County, MD, Forsyth County, NC, Jackson, MS, and suburban Minneapolis, MN) at the initial study visit (1987–1989). Participants were aged 45–64 years at enrollment and predominantly Black or White adults. Cohort examinations were conducted at visit 1 (1987–1989), visit 2 (1990–1992), visit 3 (1993–1995), visit 4 (1996–1998), visit 5 (2011–2013) and visit 6 (2016–2017). Details about the study design have been previously reported [9]. Written informed consent was obtained from all participants, and the institutional review boards at each site approved the study.

ARIC enrolled 15,027 participants. We excluded participants who had a stroke at baseline (n = 701), those who had missing data regarding stroke (n = 3478), and those who had missing data regarding other covariates of interest (n = 716). The exclusions resulted in a final sample of 10,132 participants to analyze the association between baseline TyG index and incident stroke. We also excluded participants with fewer than two valid TyG indexes during follow-up visits; the remaining 9413 participants were included in the analysis of the association between TyG index group-based trajectory and incident stroke. Data from ARIC are publicly available at the National Heart, Lung, and Blood Institute Biologic Specimen and Data Repository Information Coordinating Center and can be accessed at https://biolincc.nhlbi.nih.gov/studies/aric/.

### Data collection at baseline

Demographic data and lifestyle including age, sex, race, drinking, and smoking status were collected by self-report at visit 1 (1987–1989). Drinking and smoking status were classified as current, former, or never. Trained interviewers measured blood pressure and heart rate after a 5-minute rest. The body mass index (BMI) was calculated as weight (kg) divided by height in meters squared (m^2^). Hypertension was defined as systolic blood pressure readings ≥ 140 mmHg or diastolic blood pressure readings ≥ 90 mmHg, or the use of antihypertensive drugs in the previous two weeks. Diabetes was defined as a fasting glucose level ≥ 126 mg/dL (≥ 7.0 mmol/L), a non-fasting glucose level ≥ 200 mg/dL (≥ 11.1 mmol/L), self-reported physician diagnosis of diabetes, or any use of antidiabetic drugs. Prevalent coronary heart disease (CHD) was defined as electrocardiographic evidence of a prior myocardial infarction, or a self-reported history of a physician-diagnosed heart attack, coronary bypass surgery, or coronary angioplasty [10, 11]. Prevalent heart failure was defined as stage 3 manifest heart failure by the Gothenburg criteria or self-reported diagnosis of heart failure [12]. Peripheral artery disease (PAD) was defined as an ankle-brachial index of 0.9 or less at visit 1 [13]. Medications were ascertained through self-reported usage during the previous 2 weeks. Total cholesterol triglyceride, high-density lipoprotein cholesterol, glucose, and insulin were measured by enzymatic assays, and low-density lipoprotein cholesterol (LDL-c) was calculated using the Friedewald equation [14]. The estimated glomerular filtration rate (eGFR) was calculated using the Chronic Kidney Disease Epidemiology Collaboration creatinine equation [15]. Fibrinogen was measured from blood stored at − 70 °C using standardized protocols described previously [16]. The TyG index was calculated as

$${\text{ln}}\left( {{\text{fasting triglyceride}}\left[ {{\text{mg}}/{\text{dL}}} \right] \times {\text{fasting glucose}}\left[ {{\text{mg}}/{\text{dL}}} \right]/{\text{2}}} \right).$$

### Ascertainment of stroke events

Stroke events were identified based on hospital record reviews for identified hospitalizations and through annual phone interviews, and the methods for ascertainment are described in detail elsewhere [17]. All stroke events were identified by a computer algorithm and adjudicated by expert stroke reviewers [18]. The outcomes were definite or probable stroke events, including ischemic stroke and intracerebral hemorrhage (ICH) (excluding subarachnoid hemorrhage, due to the rare incidence) in participants without self-reporting of a physician-diagnosed stroke at baseline.

### Statistical analysis

Normally distributed continuous data were expressed as mean ± standard deviation, and the non-normally distributed continuous data were expressed as the median (interquartile range). Categorical data were expressed as numbers (percentages). Differences among groups were evaluated using analysis of variance or the Kruskal-Wallis h-test when appropriate for the continuous variables. The χ^2^ test was used for categorical variables.

Kaplan-Meier estimates were used to compute the cumulative incidence of incident stroke, ischemic stroke, and ICH by TyG index qualities. The differences were compared using the log-rank procedure. Cox proportional hazards regression model was used to calculate the hazard ratio (HR) and 95% confidence intervals (CIs) between the TyG index and the time to incident stroke. The proportional hazards assumption was assessed by graphical methods using scaled Schoenfeld residuals [19].

Three multivariate models with progressive degrees of adjustment were used to adjust for potential confounders of stroke. Model 1 was adjusted for demographic characteristics, including age, sex, and race/center (white participants, Washington County; white participants, Minneapolis; black participants, Jackson; black participants, Forsyth County; and white participants, Forsyth County). Model 2 was further adjusted for other clinical variables, including baseline smoking status, alcohol status, BMI, time-varying diabetes, time-varying PAD, and time-varying heart failure. Model 3 was further adjusted for serum parameters, physical signs and medication, including baseline systolic blood pressure, LDL-c, eGFR, fibrinogen, lipid-lowering medications and antihypertensive drugs. To determine whether our results were independent of the effects of potential drugs on the TyG index, we conducted a sensitivity analysis and repeated the analyses by excluding those participants who received any lipid- or glucose-lowering agent.

To examine the dose-response association between the TyG index with incident stroke and its subtypes, we used a restricted cubic spline regression model with three knots at the tenth, fiftieth, and ninetieth percentiles of the TyG index distribution [20]. To evaluate the robustness of the association, we performed pre-specified subgroup analyses stratifying by age, sex, race, BMI, CHD, hypertension, PAD, heart failure and diabetes at baseline. Latent profile analysis was designed to identify clusters of individuals with similar patterns of change over time [21]. We used latent class models to identify different longitudinal TyG index level patterns in ARIC Study participants who had at least two TyG index measurements during follow-up visits. We tested models with groups ranging from two to five and examined criteria including Bayesian information criteria, and determined the model with five patterns as the best fit (Additional file 1: Table S1). Participants were assigned to the trajectory group with the most significant posterior predictive probability. To estimate the association of TyG index trajectory groups with incident stroke, a trajectory group was included as an independent variable in a logistic regression model.

All analyses were conducted in R 4.0.1. A two-sided *P* value of < 0.05 was considered statistically significant.

## Results

### Baseline characteristics according to quartiles of TyG index

The mean age of all the participants was 54.1 ± 5.8 years; 46% were men. The baseline characteristics across quartiles of the baseline TyG index are displayed in Table 1. Participants with a higher baseline TyG index were older, more often male, more likely to be smokers, and had received basic education. They had a higher prevalence of hypertension, diabetes, PAD, heart failure and CHD. They more frequently used anti-hypertensive and lipid-lowering drugs. They had higher systolic and diastolic blood pressure, heart rate, BMI, fasting glucose, fasting triglyceride, insulin, total cholesterol, LDL-c, and fibrinogen but lower high-density lipoprotein cholesterol and eGFR (*P* < 0.001).

**Table 1:** Baseline characteristics of participants by quartiles of the triglyceride–glucose index

Characteristics	Total (n = 10,132)	Quartile 1 (n = 2527)	Quartile 2 (n = 2539)	Quartile 3 (n = 2532)	Quartile 4 (n = 2534)	*P* value
TyG index	8.7 ± 0.6	8.0 ± 0.2	8.4 ± 0.1	8.8 ± 0.1	9.4 ± 0.4	< 0.001
Age, years	54.1 ± 5.8	52.9 ± 5.7	53.9 ± 5.7	54.6 ± 5.7	55.1 ± 5.6	< 0.001
Male	4611 (46)	927 (37)	1070 (42)	1236 (49)	1378 (54)	< 0.001
Race-center						< 0.001
Minneapolis White participants	2661 (26)	706 (28)	679 (27)	677 (27)	599 (24)	
Jackson Black participants	2251 (22)	671 (27)	592 (23)	490 (19)	498 (20)	
Washington Co. White participants	2446 (24)	474 (19)	556 (22)	662 (26)	754 (30)	
Forsyth Co. Black participants	333 (3)	116 (5)	72 (3)	80 (3)	65 (3)	
Forsyth Co. White participants	2441 (24)	560 (22)	640 (25)	623 (25)	618 (24)	
Smoking status						< 0.001
Current smoker	2626 (26)	562 (22)	679 (27)	716 (28)	669 (26)	
Former smoker	3315 (33)	752 (30)	806 (32)	834 (33)	923 (36)	
Never smoker	4191 (41)	1213 (48)	1054 (42)	982 (39)	942 (37)	
Drinking status						< 0.001
Current drinker	5777 (57)	1503 (59)	1464 (58)	1413 (56)	1397 (55)	
Former drinker	1916 (19)	392 (16)	469 (18)	516 (20)	539 (21)	
Never drinker	2439 (24)	632 (25)	606 (24)	603 (24)	598 (24)	
Education level						< 0.001
Basic education	2240 (22)	454 (18)	538 (21)	563 (22)	685 (27)	
Intermediate education	4200 (41)	994 (39)	1035 (41)	1090 (43)	1081 (43)	
Advanced education	3692 (36)	1079 (43)	966 (38)	879 (35)	768 (30)	
Comorbidities						
Hypertension	3387 (33)	575 (23)	757 (30)	857 (34)	1198 (47)	< 0.001
Diabetes	919 (9)	42 (2)	77 (3)	131 (5)	669 (26)	< 0.001
CHD	456 (5)	53 (2)	90 (4)	113 (4)	200 (8)	< 0.001
PAD	393 (4)	78 (3)	83 (3)	112 (4)	120 (5)	0.003
Heart failure	393 (4)	45 (2)	94 (4)	90 (4)	164 (7)	< 0.001
Physical examination						
SBP, mmHg	121 ± 19.1	118 ± 19.4	120 ± 18.9	121 ± 18.1	125 ± 18.9	< 0.001
DBP, mmHg	73.3 ± 11.2	72.2 ± 11.4	72.9 ± 11.2	73.5 ± 10.9	74.7 ± 10.9	< 0.001
Heart rate, b.p.m.	66.7 ± 10.2	64.9 ± 9.64	66.0 ± 9.82	66.6 ± 10.0	69.1 ± 10.8	< 0.001
BMI, kg/m^2^	27.6 ± 5.34	25.6 ± 4.89	27.0 ± 5.25	28.1 ± 5.26	29.6 ± 5.14	< 0.001
Medication						
Anti-hypertensive medication	2915 (29)	446 (18)	645 (25)	750 (30)	1074 (42)	< 0.001
Lipid-lowering medication	321 (3)	31 (1)	76 (3)	99 (4)	115 (5)	< 0.001
Laboratory values						
Fasting glucose, mg/dL	108 ± 38.0	94.4 ± 8.61	99.3 ± 11.5	104 ± 17.5	135 ± 65.2	< 0.001
Triglyceride, mg/dL	125.±64.5	63.5 ± 12.4	94.2 ± 13.1	131 ± 20.8	209 ± 63.7	< 0.001
Insulin, µIU/mL	13.5 ± 26.0	8.31 ± 20.7	10.2 ± 12.1	13.1 ± 14.8	22.3 ± 42.4	< 0.001
TC, mg/dL	213 ± 40.9	196 ± 36.5	208 ± 37.7	219 ± 38.8	229 ± 43.1	< 0.001
LDL-c, mg/dL	137 ± 38.8	121 ± 34.7	135 ± 37.2	145 ± 37.6	145 ± 40.3	< 0.001
HDL-c, mg/dL	51.7 ± 17.0	62.9 ± 17.8	54.5 ± 15.6	47.8 ± 13.8	41.6 ± 12.5	< 0.001
eGFR, mL/min/1.73m^2^	102 ± 15.5	106 ± 15.1	103 ± 14.8	101 ± 14.8	100 ± 16.7	< 0.001
Fibrinogen, mg/dL	294 (77.0)	284 (73.0)	294 (76.0)	296 (79.0)	300 (77.0)	< 0.001
Incident stroke	909/10,132	171/2527	197/2539	243/2532	298/2534	< 0.001
Incident ischemic stroke	793/10,132	129/2527	178/2539	213/2532	273/2534	< 0.001
Incident ICH	95/10,132	29/2527	15/2539	25/2532	26/2534	0.190

Values are mean ± SD for normally distributed data and median and interquartile range for non-normally distributed data, or n (%)

*BMI*,body mass index, *CHD*,coronary heart disease, *DBP*,diastolic blood pressure, *eGFR* estimated glomerular filtration rate, *HDL-c* high-density lipoprotein cholesterol, *LDL-c*,low-density lipoprotein cholesterol, *PAD* peripheral artery disease, *SBP* systolic blood pressure, *TC* total cholesterol, *TyG* triglyceride–glucose

### Association between baseline TyG index and incident stroke

During a median follow-up of 26.6 (17.2, 28.9) years, 909 stroke cases (9.0%) were observed. As shown in Table 1, the risk of incident stroke and ischemic stroke increased with higher quartiles of the TyG index (*P* < 0.001).

In the multivariate model that measured the TyG index as a continuous variable, each unit increase in the TyG index was associated with a 32.1% higher risk of incident stroke after full adjustment for the potential confounders [HR, 1.321 (95% CI 1.161–1.504); *P* < 0.001]. Each unit increase in the TyG index was associated with a 39.8% higher risk of ischemic stroke [HR, 1.398 (95% CI 1.219–1.603); *P* < 0.001; Table 2]. The TyG index was not associated with ICH.

**Table 2:** Risk of incident stroke and its subtypes for baseline TyG index

Outcomes	TyG Index (as a categorical variable)				TyG Index (as a continuous variable)
	Quartile 1	Quartile 2	Quartile 3	Quartile 4	
Stroke					
Events/No. at risk	171/2527	197/2539	243/2532	298/2534	909/10132
Model 1	Reference	1.129 (0.920–1.387)	1.460 (1.199–1.778)**	1.876 (1.549–2.272)**	1.658 (1.486–1.849)**
Model 2	Reference	1.059 (0.862–1.302)	1.292 (1.057–1.580)*	1.441 (1.171–1.774)**	1.439 (1.269–1.633)**
Model 3	Reference	0.992 (0.805–1.221)	1.181 (0.962–1.449)	1.254 (1.014–1.552)*	1.321 (1.161–1.504)**
Ischemic stroke					
Events/No. at risk	129/2527	178/2539	213/2532	273/2534	793/10132
Model 1	Reference	1.355 (1.079–1.701)*	1.691 (1.357–2.108)**	2.276 (1.840–2.816)**	1.811 (1.614–2.033)**
Model 2	Reference	1.257 (1.001–1.579)*	1.471 (1.176–1.840)**	1.721 (1.370–2.161)**	1.527 (1.336–1.745)**
Model 3	Reference	1.169 (0.929–1.472)	1.330 (1.059–1.670)*	1.480 (1.172–1.869)*	1.398 (1.219–1.603)**
Intracerebral hemorrhage					
Events/No. at risk	29/2527	15/2539	25/2532	26/2534	95/10132
Model 1	Reference	0.530 (0.283–0.990)*	0.932 (0.543–1.600)	1.053 (0.612–1.813)	1.111 (0.774–1.595)
Model 2	Reference	0.525 (0.280–0.986)*	0.914 (0.526–1.589)	1.117 (0.622–2.005)	1.170 (0.774–1.769)
Model 3	Reference	0.494 (0.262–0.931)*	0.843 (0.479–1.484)	0.973 (0.533–1.779)	1.064 (0.695–1.629)

Model 1, adjusted for baseline age, race-center and sex

Model 2, adjusted for variables in model 1 plus baseline smoking status, alcohol status, body mass index, diabetes mellitus (time-varying), heart failure (time-varying) and peripheral artery disease (time-varying)

Model 3, adjusted for variables in model 2 plus baseline systolic blood pressure, low-density lipoprotein cholesterol, estimated glomerular filtration rate, fibrinogen, lipid-lowering drugs and antihypertensive drugs

*TyG* triglyceride–glucose

* *P* < 0.05; **P < 0.001

**Fig. 1:**
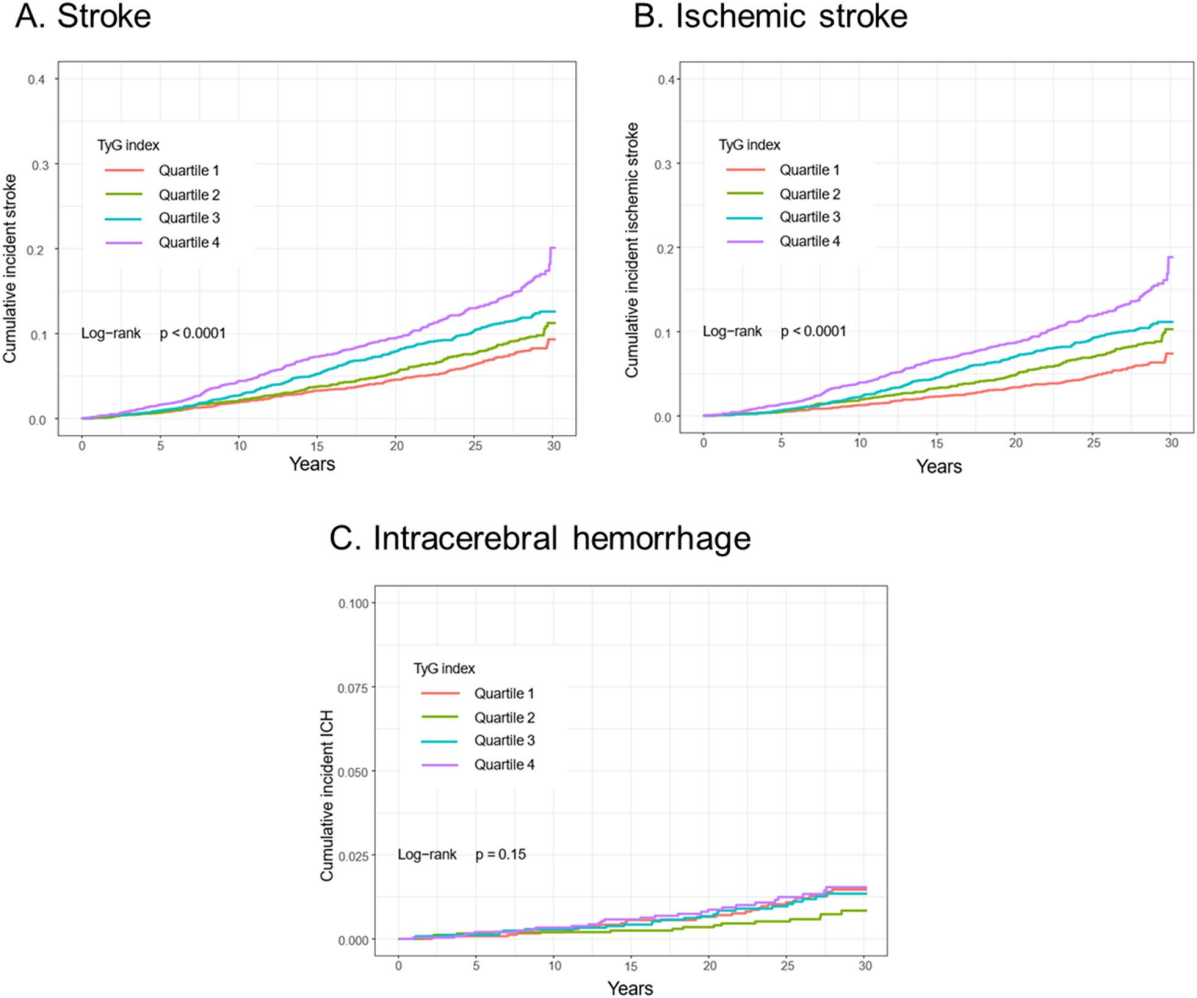
Kaplan-Meier curves of incidence of outcomes according to quartiles of baseline TyG index. **A** Stroke; **B** ischemic stroke; **C** intracerebral hemorrhage

We further categorized individuals by TyG index quartiles. Participants with higher levels of baseline TyG index had a higher risk of stroke and ischemic stroke (Fig. 1).

As shown in Table 2, the highest risk of stroke and ischemic stroke was observed in the participants with the highest TyG index quartile, in three adjusted models (all *P* < 0.05). Compared to the TyG index at quartile 1 level, quartile 2 showed lower risks of ICH in three adjusted models (all *P* < 0.05). In the sensitivity analysis that included only participants without any lipid- or glucose-lowering agent, the results were unchanged; the TyG index at the quartile 2 level showed a trend towards a lower ICH risk, though without statistical significance (Additional file 1: Table S2).

**Fig. 2:**
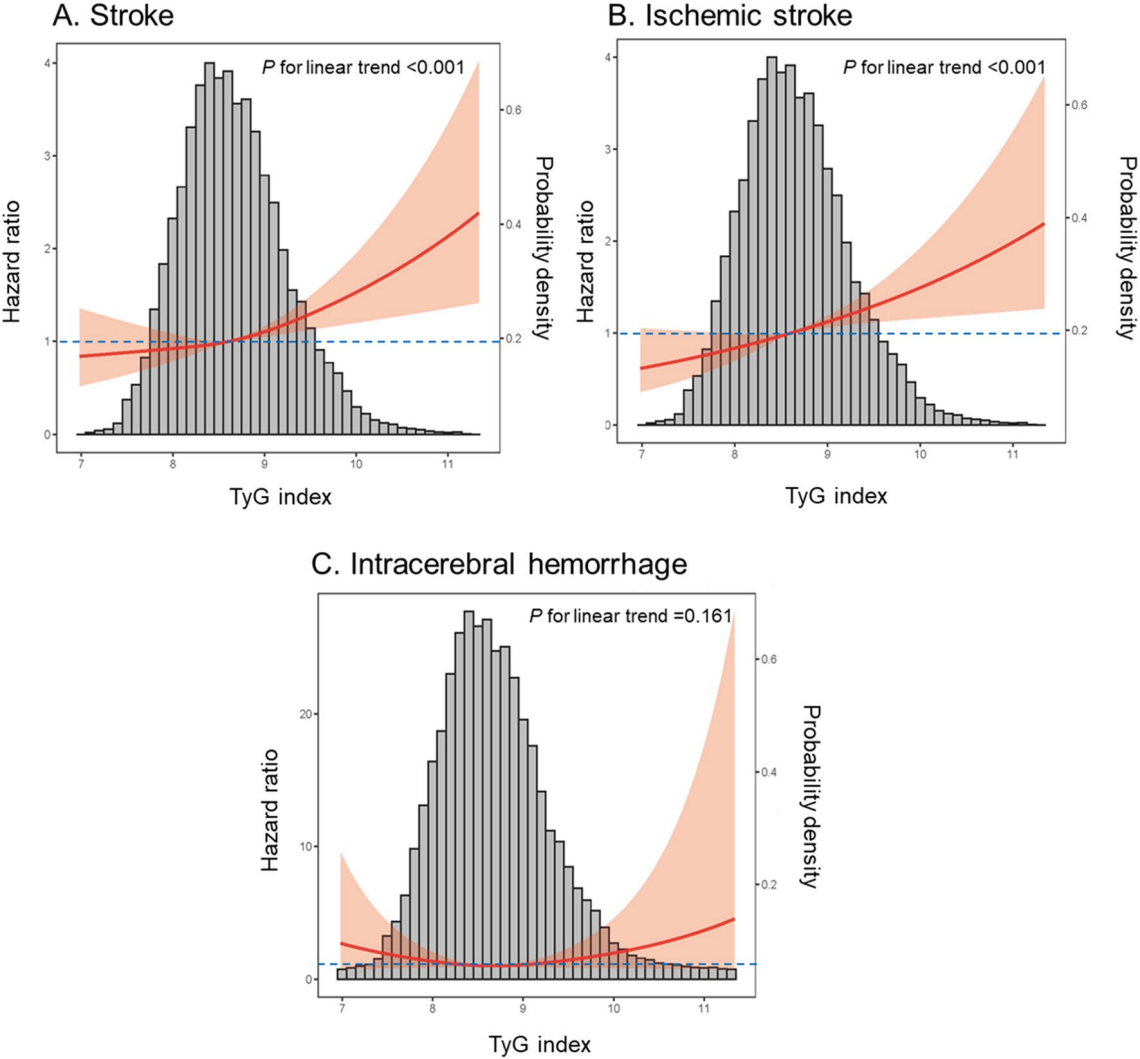
Adjusted hazard ratios of incident stroke by baseline triglyceride–glucose index. **A** Stroke; **B** ischemic stroke; **C** intracerebral hemorrhage

Figure 2 shows the restricted cubic splines of the risk of incident stroke across levels of the TyG index, suggesting linear associations between baseline TyG index levels with the risk of stroke and ischemic stroke, and non-linear associations with the risk of ICH. Consistent with the analysis using quartiles of sample distribution, the risk of total stroke and ischemic stroke increased in participants with a higher TyG index. However, there was no significant difference in the risk of total and ischemic stroke when TyG index was < 8.6 (Fig. 2).

Data were adjusted for age, race/center, sex, smoking status, alcohol status, body mass index, time-varying diabetes, time-varying peripheral artery disease, and time-varying heart failure, systolic blood pressure, low-density lipoprotein cholesterol, estimated glomerular filtration rate, fibrinogen, lipid-lowering medications and antihypertensive drugs.

Red solid line represents the hazard ratio of TyG index across the whole range. Red shadow represents the 95% CIs. Blue dotted line is the reference line as hazard ratio = 1.

When participants were stratified by age (≤ 54 or > 54 years), sex (male or female), race (white or black), smoking status (current, former or never), BMI (< 30 or ≥ 30 kg/m^2^), hypertension (yes or no), diabetes (yes or no), PAD (yes or no), heart failure (yes or no), or CHD (yes or no), the results remained consistent (Additional file 1: Figure S1). The HR (95% CI) of a higher baseline TyG index on total stroke was more prominent in participants aged < 54 years [HR = 1.382 (1.111–1.720)] than those aged ≥ 54 years [HR = 1.264 (1.075–1.485)], more prominent in the females [HR = 1.489 (1.236–1.794)] than males [HR = 1.187 (0.990–1.425)], and more prominent in the participants without CHD [HR = 1.354 (1.183–1.549)] than those with CHD [HR = 0.834 (0.521–1.337)]. Similar results of subgroup analyses were observed in the association between the TyG index and incident ischemic stroke. Subgroup analysis was not conducted in ICH patients due to the limited number of events.

### Association between TyG index trajectories and incident stroke

We included 9413 participants for trajectory analysis. Five discrete trajectories with stable TyG indexes at various levels from visits 1 to 6 were identified: low (n = 1086, 11.5%), moderate-low (n = 3186, 33.8%), moderate (n = 3164, 33.6%), moderate-high (n = 1507, 16.0%), and high (n = 470, 5.0%) (Additional file 1: Figure S2).

As shown in Additional file 1: Figure S3, the rates of incident stroke were 6.8%, 7.4%, 9.3%, 10.8%, and 16.2% in the low, moderate-low, moderate, moderate-high and high TyG index trajectory groups, respectively (*P* < 0.001). The rates of ischemic stroke were 4.9%, 6.2%, 8.5%, 9.7%, and 15.7% in the five TyG index trajectory groups, respectively (*P* < 0.001). The rates of ICH were 1.7%, 1.0%, 0.6%, 0.9%, and 0.9% in the five TyG index trajectory groups, respectively (*P* = 0.036).

Multivariate logistic regression analyses identified those with TyG index trajectories at high levels as having a greater risk of incident stroke; those at moderate, moderate-high and high levels had more significant risks of ischemic stroke, and those at moderate levels had a lower risk of ICH in three adjusted models compared with those at low level (all *P* < 0.05, Additional file 1: Table S3). In the fully adjusted model, compared with the low-level trajectory, TyG index trajectories at the high level were associated with an increasing risk of incident stroke (odds ratio [OR] 1.67, *P* = 0.010). TyG index trajectories at moderate, moderate-high, and high levels were associated with the increasing risk of ischemic stroke (OR 1.57, *P* = 0.006; OR 1.57, *P* = 0.012; OR 2.24, *P* < 0.001); TyG index trajectories at the moderate-low, moderate, moderate-high levels were associated with the lower risk of ICH (OR 0.53, *P* = 0.038; OR 0.30, *P* < 0.001; OR 0.47, *P* = 0.047, respectively) (model 3 in Additional file 1: Table S3).

## Discussion

In this large-scale, community-based prospective cohort of middle-aged US adults, we found that higher levels of baseline TyG index were significantly associated with an increased risk of stroke (particularly ischemic stroke) over a median follow-up of 27 years. We identified five distinct trajectories of the TyG index that may confer different stroke risks. A three-decade trajectory with an elevated TyG index carries a greater risk of incident stroke and ischemic stroke from baseline. Interestingly, compared with those in the lowest TyG index quartile or with a TyG index trajectory at a low level, a moderate TyG index was associated with a decreasing risk of ICH. Our study is the first to show that IR assessed by the TyG index was associated with stroke development and its subtypes among American adults.

Despite limited applicability to research settings, HIEC is the gold standard to determine IR. Several convenient IR surrogate markers have been proposed as alternatives, including the Homeostasis Model Assessment of IR (HOMA-IR), the Quantitative Insulin Sensitivity Check Index, and the McAuley index [22, 23]. The TyG index has recently become an attractive option due to the highly available and inexpensive biochemical markers needed for its calculation. Although it is uncertain which of these measures has the best diagnostic accuracy, the HOMA-IR and the TyG index are simple methods for determining IR, and the latter does not require insulin assays [24].

Previous studies showed that a higher level of the TyG index was associated with several cardiovascular diseases during long-term follow-up [25]. Evidence of the associations between the TyG index and stroke and its subtypes is limited, and most studies focused on Asians. The degree of IR [26] and the association between IR and stroke vary with race and ethnicity [7]. It is uncertain whether the results from Asians can be generalized to other races. Our study confirmed the relationship between higher TyG index levels and increased risk of stroke and ischemic stroke in a general population outside Asia. Similar to our results, a cross-sectional study of 10,900 subjects from China showed the potential value of the TyG index to optimize the risk stratification of ischemic stroke [5]. Wang et al. investigated the associations between the TyG index and stroke in a Chinese community-based cohort, suggesting that elevated baseline and long-term updated cumulative average TyG index independently predicted stroke [6]. The Rural Chinese cohort study also showed that an elevated TyG index was an independent predictor of ischemic stroke [27].

The baseline TyG index at a moderate level (mean: 8.4) decreased the risk of ICH from that of a low level (8.0). Among the identified five trajectories of the long-term TyG index, the higher level TyG index presented trends of lower risk of ICH in three adjusted models, and the moderate-low, moderate, and moderate-high level trajectory groups showed significant statistical relevance for ICH. Thus, our findings suggest that too high or too low TyG index levels may be associated with an increased risk of ICH. The association between IR and ICH remains unclear. Previous research showed that IR measured by HOMA-IR [28, 29] and HIEC [30] was not associated with ICH. A recent study showed no associations between baseline and long-term updated cumulative average TyG index and ICH in a Chinese population during an 11-year follow-up [6]. The discrepant results might be attributed to the relatively low incidence of ICH, different race characteristics, and confounders of essential risk factors for ICH such as hypertension and antithrombotic drug intake. Further research is required to confirm the association between the TyG index and ICH; nevertheless, our findings provide clues about the optimal range in which the TyG index should be controlled.

IR predicts the future risk of developing diabetes and cardiovascular diseases, and can be monitored before any signs of disease appear. Identifying the ideal cut-off values for IR markers as early as possible is crucial to reduce the risk of life-threatening diseases. The ideal cut-off value for the TyG index has not yet been entirely determined. Guerrero-Romero et al. compared the TyG index and HIEC test to evaluate their sensitivity and specificity in a small-scale cross-sectional study in Mexico, suggesting that the TyG index’s best value for the IR diagnosis was 4.68 [31]. Endukuru et al. recruited 75 metabolic syndrome patients and 75 healthy controls among southern Indian adults, and found that the ideal cutoff value for the TyG index to detect metabolic syndrome was ≥ 9.88 [32]. Moon et al. analyzed 10,997 healthy participants in Korea, and showed that the best cutoff values for metabolic abnormalities were 4.76 in men and 4.71 in women [33]. The differences in ideal cutoff points could be due to the study design, ethnic differences, sample size, or disease diagnoses. In the present study, the restricted cubic splines showed that when the TyG index is > 8.6, the risk of stroke and ischemic stroke increases with higher TyG index values. Our study may help identify the cutoff value for the TyG index to guide early treatment for cardiovascular disease prediction, especially for ischemic stroke.

Our study is the first extensive prospective study to investigate the association of baseline and the longitudinal pattern of TyG index and stroke risk among Americans. Our findings supplement the evidence that a higher TyG index is associated with an increased risk of stroke and ischemic stroke. Monitoring the TyG index requires only paired concentrations of fasting triglycerides and glucose at low prices. Risk stratification using the TyG index is inexpensive, convenient, and targets a population at high risk of stroke. Nevertheless, several limitations should be noted. First, owing to the nature of observational studies, potential residual confounders may remain; nevertheless, we carefully adjusted for various suspected risk factors and the results from different models remain consistent. Second, only middle-aged (45–64 years old) adults were recruited in the ARIC Study, and uncertain whether our results can be generalized to younger or older people. Third, though we investigated the association between the TyG index and ICH, the limited incidence may adversely affect the accuracy of the conclusion. Fourth, the temporality between TyG index and the incident stroke may be blurred in the latent profile analysis. Therefore, we cannot determine the causal relationship between five trajectories of TyG index and stroke. Finally, although the TyG index substantially correlated with the gold standard HIEC test and was a reliable indicator of IR in previous studies, we could not evaluate the correlation between the TyG index and the HIEC test, which was not performed in the ARIC Study.

## Conclusions

Elevated l baseline and long-term TyG index levels are associated with an increased risk of stroke, especially ischemic stroke. Too high or too low TyG index levels may be associated with an increased risk of ICH. The results support the contribution of the TyG index to the development of stroke and its subtypes in American adults. Enhanced monitoring of the TyG index is needed to prevent and reduce the occurrence of stroke.

### Supplementary Information

**Additional file 1: Table S1. **Group-based trajectory model fit summary (N=9413). **Table S2. **Risk of incident stroke for baseline TyG index among participants without any lipid- or glucose-lowering medication. **Figure S1. **Subgroup analysis of the association between baseline TyG index and stroke. **Figure S2.** Trajectories by TyG index in the Atherosclerosis Risk in Communities Study. **Figure S3.** Prevalence of incident stroke and its subtypes across the triglyceride-glucose index trajectory groups. **Table S3.** Risk of incident stroke and its subtypes for different levels of triglyceride-glucose index trajectory groups

## Data Availability

The dataset supporting the conclusions of this article is available in the BioLINCC website, [https://biolincc.nhlbi.nih.gov/studies/?q=ARIC].
