# Supplementary material for: Triglyceride–glucose index and the risk of stroke in American adults: findings from the atherosclerosis risk in communities study
Source: Diabetol Metab Syndr. 2023 Sep 19;15:187. doi: 10.1186/s13098-023-01161-3 (PMC10507886; doi:10.1186/s13098-023-01161-3)
Supplement: Supplementary file 1 — Additional file 1: Table S1. Group-based trajectory model fit summary (N=9413). Table S2. Risk of incident stroke for baseline TyG index among participants without any lipid- or glucose-lowering medication. Figure S1. Subgroup analysis of the association between baseline TyG index and stroke. Figure S2. Trajectories by TyG index in the Atherosclerosis Risk in Communities Study. Figure S3. Prevalence of incident stroke and its subtypes across the triglyceride-glucose index trajectory groups. Table S3. Risk of incident stroke and its subtypes for different levels of triglyceride-glucose index trajectory groups [file 13098_2023_1161_MOESM1_ESM.docx]

**Additional materials**

**Table S1** Group-based trajectory model fit summary (N=9413).

| Model | AIC | BIC | SABIC | Latent class probabilities |
| --- | --- | --- | --- | --- |
| 2 | 77916.539 | 78052.388 | 77992.009 | 0.364/0.636 |
| 3 | 70220.804 | 70406.703 | 70324.079 | 0.156/ 0.342/0.502 |
| 4 | 66268.799 | 66504.747 | 66399.879 | 0.067/0.228/0.260/0.446 |
| 5 | 65090.296 | 65376.294 | 65249.180 | 0.050/0.115/0.160/0.336/0.338 |

*AIC* Akaike’s information criterion, *BIC* Bayesian information criterion, *SABIC* sample-adjusted Bayesian information criterion.

**Table S2** Risk of incident stroke for baseline TyG index among participants without any lipid- or glucose-lowering medication

| Outcomes | TyG Index (as a categorical variable) | | | | TyG Index  (as a continuous variable) |
| --- | --- | --- | --- | --- | --- |
|  | Quartile 1 | Quartile 2 | Quartile 3 | Quartile 4 |  |
| Stroke | | | | | |
| Events/No. at risk | 99/1814 | 131/1818 | 157/1816 | 167/1815 | 554/7260(0.757) |
| Model 1 | Reference | 1.243(0.956-1.616) | 1.622(1.257-2.094)** | 1.897(1.468-2.450)** | 1.828(1.550-2.156)** |
| Model 2 | Reference | 1.174(0.902-1.528) | 1.446(1.116-1.873)* | 1.530(1.172-1.999)* | 1.652(1.389-1.964)** |
| Model 3 | Reference | 1.108(0.850-1.444) | 1.353(1.040-1.761)* | 1.307(0.993-1.720) | 1.435(1.203-1.713)** |
| Ischemic stroke | | | | | |
| Events/No. at risk | 68/1814 | 113/1818 | 130/1816 | 146/1815 | 457/7260(0.779) |
| Model 1 | Reference | 1.556(1.150-2.105)* | 1.929(1.434-2.596)** | 2.371(1.764-3.186)** | 1.982(1.655-2.374)** |
| Model 2 | Reference | 1.456(1.075-1.972)* | 1.709(1.265-2.307)** | 2.007(1.481-2.720)** | 1.755(1.452-2.121)** |
| Model 3 | Reference | 1.368(1.008-1.855)* | 1.571(1.158-2.132)* | 1.666(1.219-2.276)* | 1.503(1.238-1.824)** |
| Intracerebral hemorrhage | | | | | |
| Events/No. at risk | 21/1814 | 13/1818 | 21/1816 | 19/1815 | 74/7260(0.707) |
| Model 1 | Reference | 0.617(0.308-1.238) | 1.148(0.620-2.125) | 1.214(0.636-2.316) | 1.678(1.061-2.654)* |
| Model 2 | Reference | 0.613(0.305-1.233) | 1.082(0.577-2.030) | 1.157(0.593-2.258) | 1.639(1.015-2.646)* |
| Model 3 | Reference | 0.573(0.284-1.156) | 1.016(0.534-1.930) | 0.999(0.500-1.994) | 1.472(0.906-2.392) |

Model 1, adjusted for baseline age, race-center and sex

Model 2, adjusted for variables in model 1 plus baseline smoking status, alcohol status, body mass index, diabetes mellitus (time-varying), heart failure (time-varying) and peripheral artery disease (time-varying)

Model 3, adjusted for variables in model 2 plus baseline systolic blood pressure, low-density lipoprotein cholesterol, estimated glomerular filtration rate, fibrinogen, lipid-lowering drugs and antihypertensive drugs

TyG, triglyceride-glucose

**P*<0.05; ***P*<0.001

A. Total stroke


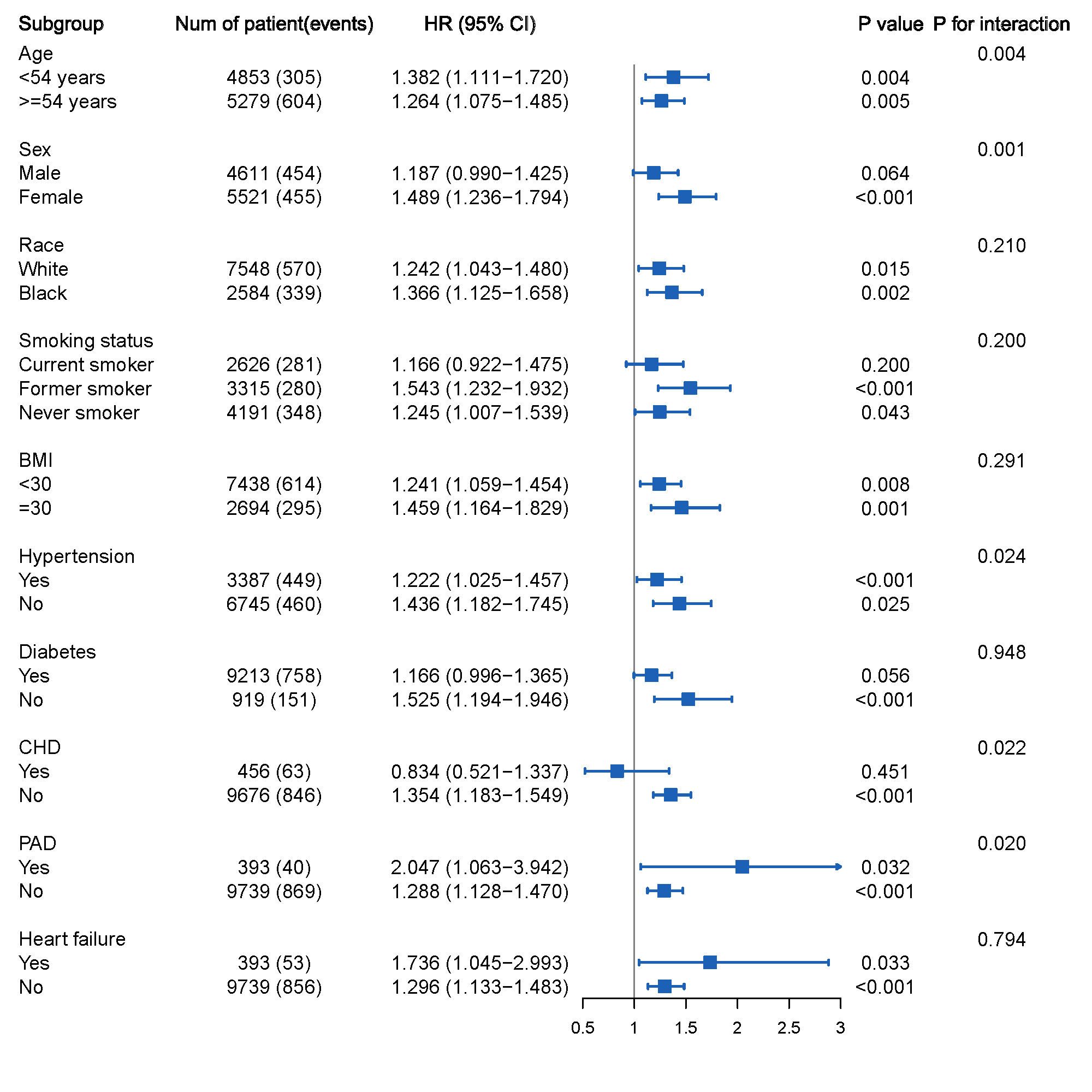


B. Ischemic stroke


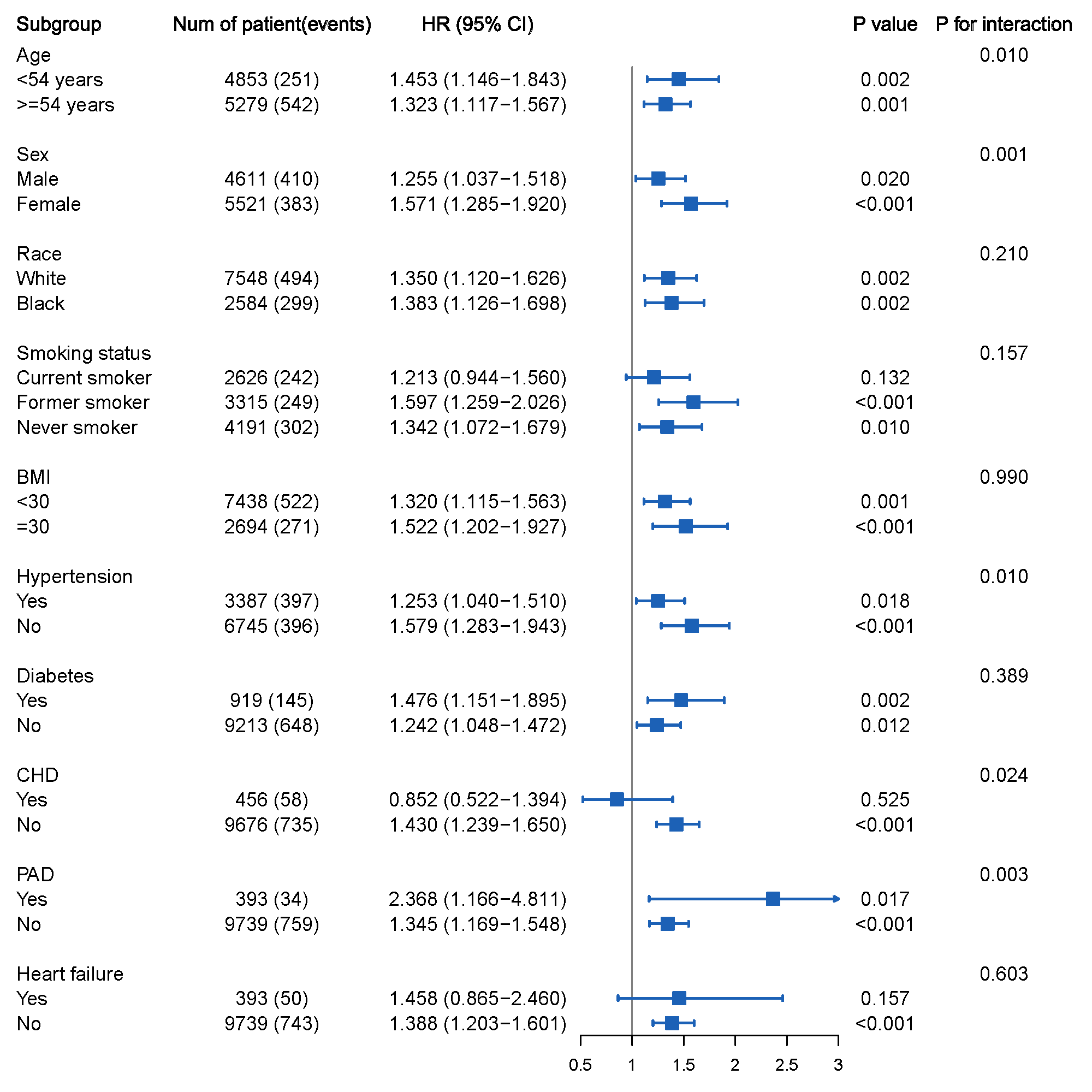


**Figure S1** Subgroup analysis of the association between baseline TyG index and stroke. A. total stroke, B. ischemic stroke.

Cox regression after adjustment for age, race-center, sex, smoking status, alcohol status, body mass index, diabetes mellitus, peripheral artery disease, heart failure, systolic blood pressure, low-density lipoprotein cholesterol, estimated glomerular filtration rate, fibrinogen, lipid-lowering drugs and antihypertensive drugs was performed in subgroups according to age (≤ 54 or > 54 years), sex (male or female), race (White or black), smoking status (current or former or never), body mass index (BMI; < 30 or ≥ 30 kg/m2), hypertension (yes or no), diabetes (yes or no) , peripheral artery disease (yes or no), heart failure (yes or no), and coronary heart disease (yes or no).

BMI, body mass index; CHD, coronary heart disease; PAD, peripheral artery disease


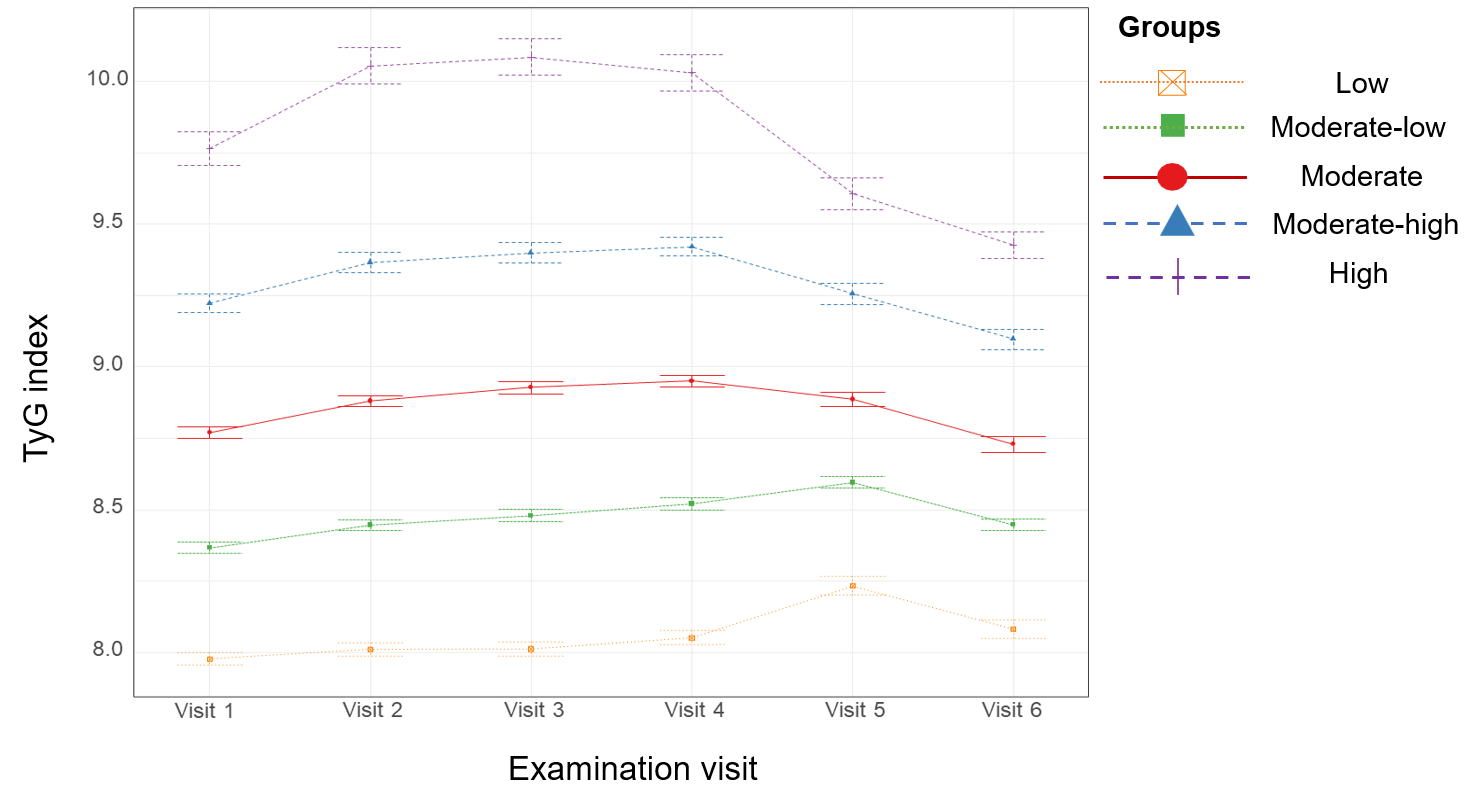


**Figure S2** Trajectories by TyG index in the Atherosclerosis Risk in Communities Study

**
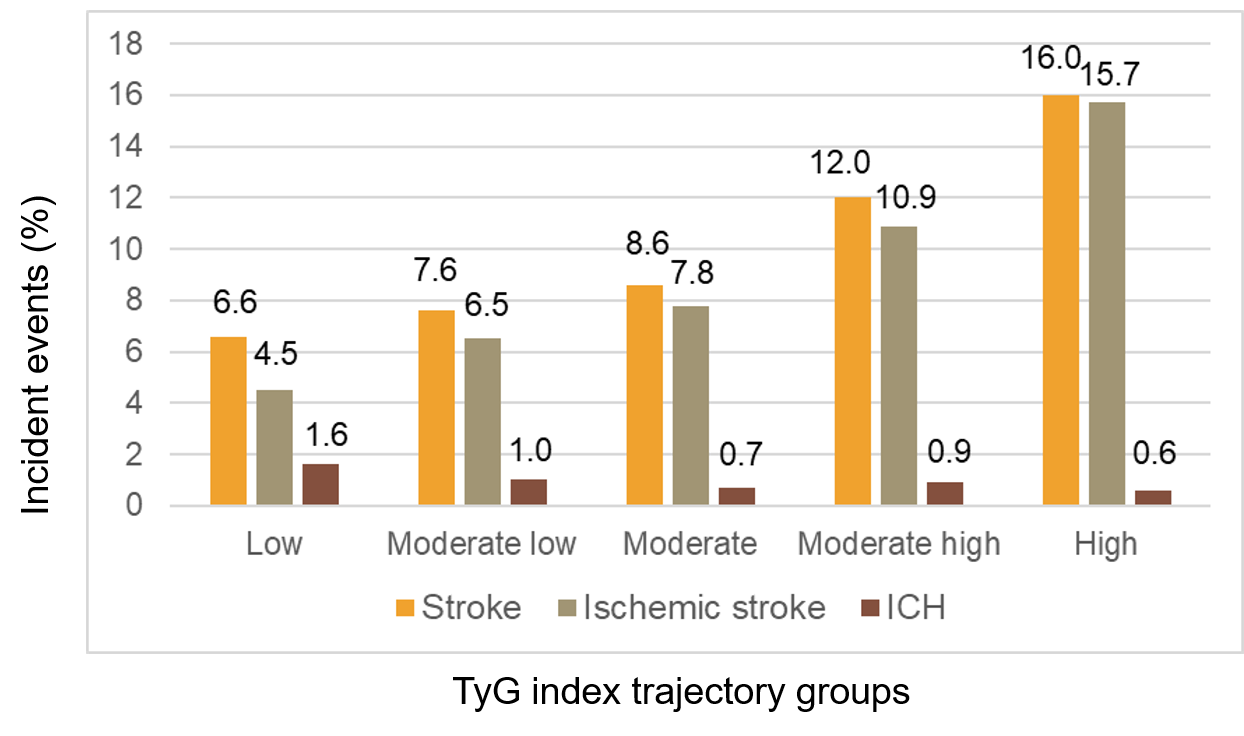
**

**Figure S3** Prevalence of incident stroke and its subtypes across the triglyceride-glucose index trajectory groups

ICH, intracerebral hemorrhage

**Table S3** Risk of incident stroke and its subtypes for different levels of triglyceride-glucose index trajectory groups

| TyG index trajectories | Model 1  OR (95CI%) | *P* value | Model 2  OR (95CI%) | *P* value | Model 3  OR (95CI%) | *P* value |
| --- | --- | --- | --- | --- | --- | --- |
| Stroke | | | | | | |
| Low | Reference | 1.0 | Reference | 1.0 | Reference | 1.0 |
| Moderate-low | 1.11(0.85-1.47) | 0.443 | 1.07(0.81-1.42) | 0.638 | 1.02(0.77-1.35) | 0.904 |
| Moderate | **1.44(1.10-1.89)** | **0.008** | 1.31(1.00-1.74) | 0.051 | 1.20(0.91-1.59) | 0.211 |
| Moderate-high | **1.65(1.24-2.22)** | **<0.001** | **1.43(1.05-1.94)** | **0.023** | 1.26(0.93-1.73) | 0.146 |
| High | **2.44(1.73-3.45)** | **<0.001** | **1.89(1.29-2.78)** | **0.001** | **1.67(1.13-2.48)** | **0.010** |
| Ischemic stroke | | | | | | |
| Low | Reference | 1.0 | Reference | 1.0 | Reference | 1.0 |
| Moderate-low | 1.36(1.00-1.88) | 0.059 | 1.30(0.95-1.80) | 0.111 | 1.23(0.90-1.71) | 0.202 |
| Moderate | **1.92(1.42-2.64)** | **<0.001** | **1.73(1.27-2.39)** | **<0.001** | **1.57(1.15-2.18)** | **0.006** |
| Moderate-high | **2.14(1.54-3.00)** | **<0.001** | **1.78(1.27-2.54)** | **0.001** | **1.57(1.11-2.25)** | **0.012** |
| High | **3.45(2.37-5.07)** | **<0.001** | **2.54(1.67-3.87)** | **<0.001** | **2.24(1.47-3.43)** | **<0.001** |
| Intracerebral hemorrhage | | | | | | |
| Low | Reference | 1.0 | Reference | 1.0 | Reference | 1.0 |
| Moderate-low | 0.61(0.34-1.12) | 0.10 | 0.57(0.32-1.05) | 0.063 | **0.53(0.30-0.99)** | **0.038** |
| Moderate | **0.38(0.19-0.73)** | **0.003** | **0.34(0.17-0.68)** | **0.002** | **0.30(0.15-0.60)** | **<0.001** |
| Moderate-high | 0.57(0.28-1.17) | 0.126 | 0.55(0.25-1.16) | 0.117 | **0.45(0.21-0.98)** | **0.047** |
| High | 0.49(0.14-1.33) | 0.200 | 0.49(0.13-1.54) | 0.258 | 0.42(0.11-1.33) | 0.170 |

Model 1, adjusted for baseline age, race-center and sex

Model 2, adjusted for variables in model 1 plus baseline smoking status, alcohol status, body mass index, diabetes mellitus, heart failure and peripheral artery disease

Model 3, adjusted for variables in model 2 plus baseline systolic blood pressure, low-density lipoprotein cholesterol, estimated glomerular filtration rate, fibrinogen, lipid-lowering drugs and antihypertensive drugs

CIs, confident intervals; OR, odds ratio; TyG, triglyceride-glucose
